# Decreased vasoregulatory dysfunction associated with intra-operative hemoadsorption treatment is related to mitigated post-transplant procalcitonin rather than cytokine or complement response

**DOI:** 10.3389/fmed.2025.1541519

**Published:** 2025-08-13

**Authors:** Hajna Katona, Adam Soltesz, Eniko Kovacs, Zsofia Szakal-Toth, Eszter Tamaska, Kristof Racz, Tamás Radovits, Attila Fintha, Krisztián Kovács, Lisa Hurler, Zoltán Prohászka, Bela Merkely, Endre Nemeth

**Affiliations:** ^1^Heart and Vascular Center, Semmelweis University, Budapest, Hungary; ^2^University Department of Anesthesiology and Intensive Therapy, Semmelweis University, Budapest, Hungary; ^3^Department of Pathology and Experimental Cancer Research, Semmelweis University, Budapest, Hungary; ^4^Department of Laboratory Medicine, Semmelweis University, Budapest, Hungary; ^5^Department of Internal Medicine and Hematology, Semmelweis University, Budapest, Hungary

**Keywords:** complement system, cytokines, CytoSorb, hemoadsorption, heart transplantation, procalcitonin, vasopressor score

## Abstract

**Introduction:**

The aim of this study was to investigate the modulatory effect of intraoperative hemoadsorption (HA) versus standard care on the perioperative inflammatory profile of patients undergoing orthotopic heart transplantation (OHT) and its correlation with the severity of post-transplant vasoregulatory dysfunction.

**Methods:**

In this secondary analysis, data from NCT03145441, a prospective, single-center, open-label, randomized controlled trial were used.

**Results:**

Patients in the HA group had a lower median vasopressor score, rate of severe vasoregulatory dysfunction (vasopressor score: 19.7 [7.9–37.8] vs. 35.6 [14.5–57.6], *p* = 0.031, respectively and severe vasoregulatory dysfunction: 33.3% vs. 60.0% *p* = 0.048, respectively), and reduced procalcitonin (PCT) level (PCT: 0.93 [0.38–2.36] μg/L vs. 2.08 [1.13–6.36] μg/L, *p* = 0.007, respectively) during the early postoperative period than patients in the control group. The 24-h cytokine and complement levels were comparable in the study groups. The 24-h inflammatory profile of HA and control groups remained unchanged in the cluster of severe vasoregulatory dysfunction. There was a moderate positive correlation between the vasopressor score and the PCT concentration in the control group (*r*_S_: 0.605, *p* = 0.002) which was not identified in the HA group.

**Discussion:**

Intraoperative HA treatment exerts a beneficial modulatory effect on the postoperative PCT response in OHT recipients, which is directly associated with significantly lower rates of post-transplant severe vasoregulatory dysfunction compared to controls.

## 1 Introduction

Severe vasoregulatory dysfunction, which may result in vasoplegic syndrome (VS), is a common occurrence during the perioperative period following orthotopic heart transplantation (OHT) (1, 2). This complication, in conjunction with the excessive use of vasopressors, contributes significantly to the development of post-transplant multiorgan dysfunction and elevates recipients’ risk for adverse outcomes (2). Although a standardized definition of VS is currently lacking in the literature, the presented coherent cut-off values for vasopressors indicate that clinically relevant vasoregulatory dysfunction may occur in over 50% of OHT recipients (1, 2). Despite the significant number of previous clinical investigations on OHT-related vasoregulatory dysfunction, the complex pathophysiological environment that underlies this phenomenon remains unclear (3).

The severe vasoregulatory dysfunction associated with OHT often fails to respond to high doses of vasopressors, and to date there is no other specific pharmacological treatment that has been demonstrated to be effective in controlling or preventing VS (4). However, the results of the most recent proof-of-concept randomized controlled trial (RCT) from our research group suggest better post-transplant hemodynamic stability, as confirmed by a significantly lower vasoactive-inotropic score in recipients who received intraoperative hemoadsorption (HA) treatment, a non-pharmacological blood purification technology, compared to controls (5). In recent years, a number of clinical studies have focused on the perioperative characteristics of various inflammatory markers, including cytokines, C-reactive protein and procalcitonin, in the context of cardiac surgery and intraoperative HA (6). Nevertheless, there is considerable methodological diversity in the measurement of inflammatory markers, and the presented results regarding the modulatory effects of HA on the perioperative inflammatory response are controversial (6). Interestingly, none of the aforementioned studies included patients from the OHT subgroup.

In comparison to non-transplant cardiac surgeries, OHT is known to be associated with more complex inflammatory responses utilizing a distinctive network of immune cells and related cytokines and chemokines, which are partially influenced by the induction of exogenous immunosuppression (7, 8). The OHT-associated complex inflammatory response is primarily determined by the actual preoperative baseline pro-inflammatory balance as part of the pathophysiology of advanced chronic heart failure (9, 10). A wide range of cytokines, including tumor necrosis factor alpha (TNF-α), interleukin– (IL) IL-1β, IL-6, IL-8, IL-10, and IL-18, are involved in this persistent low-grade inflammation, which plays a pivotal role in the consecutive endothelial injury (10, 11). This, in turn, has been demonstrated to promote the chronic disturbance of vascular tone and the increased sensitivity of the endothelium to oxidative stress (11). The subsequent triggers of the OHT-associated perioperative inflammatory response may emerge from the prolonged total ischemic time of the cardiac allograft, cardiopulmonary bypass (CPB) time and the solid organ transplantation induced host immune response (2, 12). These interact with chronically activated inflammation, with the potential to exaggerate the early post-transplant inflammatory response. Accordingly, detailed analysis of the relationship between the OHT-induced inflammatory response and post-transplant vasoregulatory dysfunction can be important in clarifying the potential role of the intraoperative HA in their modulation, with clinical data in this field currently absent. The aim of this study was to use data from our proof-of-concept RCT to investigate the modulatory effect of intraoperative HA on the perioperative inflammatory profile of OHT and its correlation with the severity of post-transplant vasoregulatory dysfunction.

## 2 Materials and methods

### 2.1 Study design

This study used data from our prospective, single-center, open-label, randomized controlled trial (RCT); ClinicalTrials.gov identifier: NCT03145441, which has been described in detail previously (5). Briefly, this RCT was done in compliance with the Declaration of Helsinki and approved by the Semmelweis University Regional and Institutional Committee of Science and Research Ethics. Written informed consent was obtained from all participants. A total of 60 adult OHT candidates registered on the waiting list (aged ≥ 18 years) with United Network for Organ Sharing status 6 were included in the study (5). According to the randomization scheme, participants were allocated to receive intraoperative hemoadsorption (HA group *N* = 30) or standard care (control group *N* = 25 plus 5 exclusions) (5).

### 2.2 Intraoperative hemoadsorption treatment

The management of patients in the perioperative period has been reported previously (5). Briefly, all patients enrolled in this study received standardized anesthetic, surgical and post-operative intensive care according to institutional protocol, including hemodynamic and hemostasis management, immunosuppressive treatment and follow-up examinations. Patients randomly assigned to the HA group received standard OHT care in conjunction with intraoperative HA, utilizing a CytoSorb™ 300 mL cartridge (CytoSorbents™, Monmouth Junction, NJ, United States) for a one-cycle treatment throughout the entire period of CPB (5).

### 2.3 Inflammatory biomarker measurements

All inflammatory biomarkers were quantified from venous blood samples collected at designated time points, in accordance with the study protocol. The separation of serum and plasma from native and k3-EDTA-anticoagulated blood samples, respectively, was conducted by centrifugation (3,500 rpm, 10 min, room temperature). The samples were then divided into 0.5 mL screw cap microtubes (Sarstedt AG & Co. KG, Nuembrecht, Germany) and stored at −80°C. TNF-α, IL-6, IL-1β, and IL-10 levels were measured using the Legend Max quantitative ELISA assay (BioLegend, San Diego, CA, United States). The complement activation markers of C3a and C4a concentrations were quantified by MicroVue ELISA kits (QuidelOrtho, San Diego, CA, United States) and sC5b-9 (terminal complement complex, TCC) was quantified by TCC ELISA kit (HyCult Biotech, Uden, The Netherlands). The levels of C-reactive protein (CRP) and procalcitonin (PCT) were determined using standardized, validated laboratory measurements.

### 2.4 Outcomes

The primary endpoint of the original proof-of-concept RCT has been previously presented (5). In this secondary analysis, the outcomes were the severity of postoperative vasoregulatory dysfunction as assessed by the vasopressor score (vasopressor score = [adrenaline dose × 100 μg/kg/min] + [noradrenaline dose × 100 μg/kg/min] + [vasopressin dose × 10,000 units/kg/min] (1), based on the mean doses used in the first 24 h postoperatively for each adjusted agent), characteristics of the early postoperative inflammatory response described by changes in TNF-α, IL-6, IL-1β, IL-10, C3a, C4a, TCC, CRP, and PCT and their correlation with the severity of vasoregulatory dysfunction. The vasopressor score was considered ‘high’ if it was >30 points, indicating a patient group at high risk of adverse outcomes.

### 2.5 Statistical analysis

All statistical tests were performed with IBM SPSS Statistics for Windows, version 28.0.1.0 (IBM Corp., Armonk, NY, United States). Continuous variables were tested for normality using the Shapiro–Wilk test. The descriptive statistics of the measured parameters were presented as median [interquartile range], mean ± standard deviation, and number of patients and frequency, where appropriate. The univariate analysis of group comparisons was conducted using the Mann–Whitney U test, two-sample *t*-test, χ^2^ test or Fisher’s exact test. The Wilcoxon signed-rank test was used for the comparative analysis of within-subject changes in the cohort. Spearman’s correlations were employed to ascertain any potential association between vasopressor score and specified inflammatory markers elucidating their possible link with the severity of postoperative vasoregulatory dysfunction. In all tests, the threshold for statistical significance was set at a *P*-value of 0.05.

## 3 Results

### 3.1 Severity of vasoregulatory dysfunction

**Figure 1:**
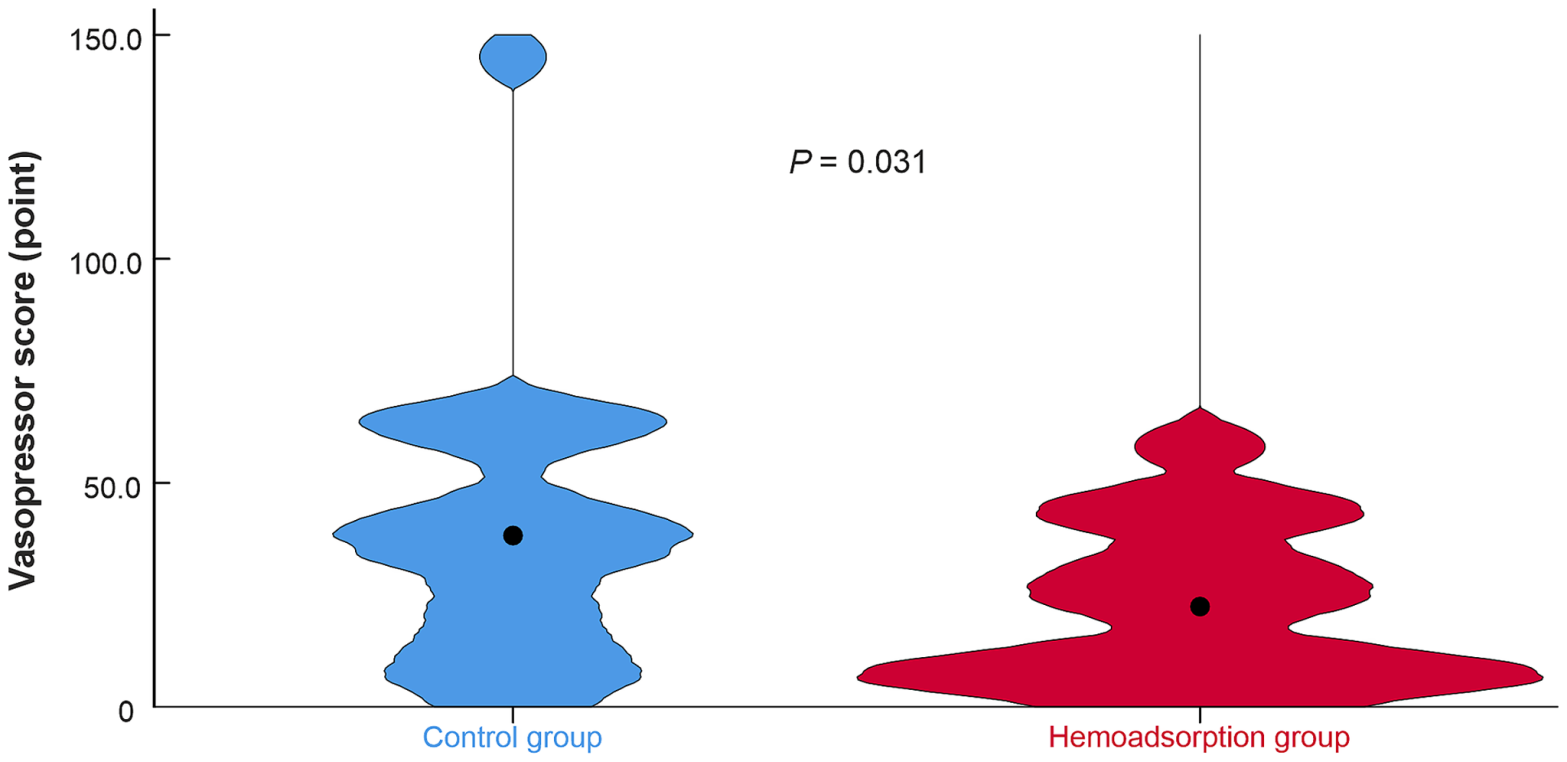
Distribution of the post-transplant vasopressor score in the study groups. Violin plot, *N* = 55. Filled circle indicates group median.

A total of 60 patients were randomly assigned to either the control group (*N* = 30) or the HA group (*N* = 30) during the study period. Five patients from the control group were excluded from the primary outcome analyzes (5). The patients who received intraoperative hemoadsorption treatment exhibited a markedly reduced vasopressor score in comparison to the control group during the initial 24-h postoperative period (median vasopressor score: 19.7 [7.9–37.8] vs. 35.6 [14.5–57.6], *p* = 0.031, respectively). The distribution of the vasopressor scores in the HA and control groups are displayed in Figure 1. In accordance with the definition of severe vasoregulatory dysfunction, its observed frequency was 33.3% (10 patients) in the HA group and 60.0% (15 patients) in the control group (*p* = 0.048).

### 3.2 Characteristics of the early postoperative inflammatory response

The preoperative profiles of cytokines, complements, CRP (5) and PCT were found to be comparable between the study groups (Table 1). Furthermore, the baseline IL-6 level, and IL-6/IL-10 ratio were markedly lower in the HA group compared to the control group. Nevertheless, the IL-6 levels were within the clinical reference range, and the IL-6/IL-10 ratios were found to be less than 1.0 in the study groups. Among the inflammatory markers, CRP, PCT, IL-6, and IL-10 demonstrated a statistically significant increase at 24 h post-CPB in both the HA and control groups (*p* < 0.001). In contrast, TNF-α, IL-1β, C3a, and TCC did not exhibit any change over this time period. A statistically significant reduction in C4a was observed in the control group (*p* = 0.021), whereas no change was noted in the HA group. The between-group comparisons revealed no significant differences in CRP, cytokines and complements 24 h after the CPB start, and the IL-6/IL-10 ratios remained within the range of <1.0 (Table 2). Conversely, the PCT levels exhibited a significantly lower median concentration in the HA group relative to the control group at this measurement point (*p* = 0.007, Table 2). Furthermore, our findings revealed that the peak value of PCT > 1.0 μg/L was observed in 50.0% of subjects in the HA group (15 patients) compared to 84.0% (21 patients) of controls (*p* = 0.011). The subgroups of selected patients defined as having a vasopressor score >30 did not exhibit any differences in their early postoperative inflammatory profile, with the exception of the PCT response. Accordingly, both the PCT level and the rate of peak PCT > 1.0 μg/L were significantly lower in the selected HA-treated patients compared to the selected controls (PCT: 0.98 [0.44–3.33] μg/L vs. 5.72 [1.31–7.19] μg/L, *p* = 0.033 and PCT > 1.0 μg/L: 50.0% (5 patients) vs. 93.3% (14 patients), *p* = 0.023, respectively). Table 3 summarizes the characteristics of the early postoperative inflammatory response in the cluster of patients who developed severe vasoregulatory dysfunction.

**Table 1:** Baseline inflammatory characteristics of the study population prior to heart transplantation.

Inflammatory markers	Control group (*N* = 25)	Hemoadsorption group (*N* = 30)	*P*-value
CRP, mg/L	3.3 [1.8–7.3]	2.3 [0.9–4.8]	0.151
PCT, μg/L	0.04 [0.03–0.09]	0.04 [0.02–0.07]	0.463
TNF-α, pg./mL	0.88 [0.07–10.98]	0.25 [0.07–9.29]	0.764
IL-6, pg./mL	4.55 [2.21–16.10]	2.88 [0.01–5.72]	0.029
IL-1β, pg./mL	2.17 [0.43–5.26]	3.55 [0.74–10.09]	0.122
IL-10, pg./mL	6.89 [4.14–13.80]	8.14 [4.49–12.52]	0.600
IL-6/IL–10 ratio	0.80 [0.19–3.10]	0.30 [0.001–1.22]	0.048
C3a, ng/mL	163.4 [97.3–272.9]	119.95 [82.13–182.43]	0.166
C4a, ng/mL	693.4 [537.1–1075.2]	554.9 [438.2–715.4]	0.051
TCC, mAu/mL	2903.2 ± 648.5	2595.7 ± 851.9	0.144

CRP, C-reactive protein; PCT, procalcitonin; TNF-α, tumor necrosis factor alpha; IL-6, interleukin-6; IL-1β, interleukin-1 beta; IL-10, interleukin-10; C3a, complement 3a; C4a, complement 4a; TCC, terminal complement complex.

**Table 2:** Inflammatory characteristics measured 24 h after CPB start.

Inflammatory markers	Control group (*N* = 25)	Hemoadsorption group (*N* = 30)	*P*-value
CRP, mg/L	86.6 [51.3–128.1]	79.9 [30.5–95.1]	0.108
PCT, μg/L	2.08 [1.13–6.36]	0.93 [0.38–2.36]	0.007
TNF-α, pg./mL	0.07 [0.07–5.77]	0.07 [0.07–1.07]	0.681
IL-6, pg./mL	82.8 [51.6–139.3]	72.3 [48.8–171.0]	0.822
IL-1β, pg./mL	1.06 [0.38–3.68]	4.22 [0.95–7.05]	0.058
IL-10, pg./mL	144.9 [48.2–250.0]	106.7 [56.8–240.4]	0.896
IL-6/IL–10 ratio	0.78 [0.36–1.44]	0.70 [0.34–1.74]	0.801
C3a, ng/mL	140.2 [105.3–225.9]	131.1 [84.0–211.3]	0.286
C4a, ng/mL	509.5 [389.2–750.1]	538.2 [376.4–654.9]	0.815
TCC, mAu/mL	3008.1 ± 1310.2	2653.6 ± 497.2	0.183

CRP, C-reactive protein; PCT, procalcitonin; TNF-α, tumor necrosis factor alpha; IL-6, interleukin-6; IL-1β, interleukin-1 beta; IL-10, interleukin-10; C3a, complement 3a; C4a, complement 4a; TCC, terminal complement complex.

**Table 3:** Inflammatory characteristics in a cluster of patients developed severe vasoregulatory dysfunction (vasopressor score > 30) measured 24 h after CPB start.

Inflammatory markers	Control group (*N* = 15)	Hemoadsorption group (*N* = 10)	*P*-value
CRP, mg/L	78.1 [35.5–118.3]	77.9 [29.3–119.3]	0.657
PCT, μg/L	5.72 [1.31–7.19]	0.98 [0.44–3.33]	0.033
TNF-α, pg./mL	0.07 [0.07–0.07]	0.07 [0.07–0.07]	0.401
IL-6, pg./mL	79.3 [41.7–139.9]	100.5 [47.1–167.4]	0.560
IL-1β, pg./mL	0.95 [0.21–1.66]	5.92 [0.43–10.43]	0.075
IL-10, pg./mL	194.5 [48.4–250.0]	220.1 [86.1–245.4]	0.866
IL-6/IL–10 ratio	0.64 [0.33–1.23]	0.52 [0.38–1.44]	0.912
C3a, ng/mL	174.8 [100.5–239.0]	155.2 [90.8–209.1]	0.506
C4a, ng/mL	509.5 [385.5–901.3]	491.2 [351.0–646.9]	0.506
TCC, mAu/mL	3076.1 ± 1679.7	2817.9 ± 370.6	0.639

CRP, C-reactive protein; PCT, procalcitonin; TNF-α, tumor necrosis factor alpha; IL-6, interleukin-6; IL-1β, interleukin-1 beta; IL-10, interleukin-10; C3a, complement 3a; C4a, complement 4a; TCC, terminal complement complex.

### 3.3 Spearman correlation between the measured inflammatory markers and vasopressor score

The Spearman correlation analysis did not identify a statistically significant association between the 24-h post-CPB CRP, cytokine, and complement levels and vasopressor scores in the study groups. Nevertheless, a notable correlation was observed between the early postoperative PCT concentrations and vasopressor scores in the control group (*r*_S_: 0.605, *p* = 0.002, Table 4). This relationship was not seen in the HA group. The results of the Spearman correlation analysis are presented in Table 4.

**Table 4:** Association between the vasopressor score and inflammatory markers.

Inflammatory markers	Vasopressor score					
	Control group (*N* = 25)			Hemoadsorption group (*N* = 30)		
	Spearman’s rho	95% CI	*P*-value	Spearman’s rho	95% CI	*P*-value
CRP	−0.162	−0.532 to 0.261	0.440	0.131	−0.251 to 0.478	0.489
PCT	0.605	0.255–0.815	0.002	0.312	−0.066 to 0.611	0.094
TNF-α	−0.215	−0.570 to 0.209	0.303	0.025	−0.355 to 0.398	0.897
IL-6	0.083	−0.334 to 0.472	0.694	0.137	−0.253 to 0.488	0.480
IL-1β	−0.481	−0.742 to 0.093	0.015	−0.038	−0.408 to 0.343	0.845
IL-10	0.275	−0.147 to 0.612	0.184	0.335	−0.047 to 0.632	0.076
IL-6/IL-10 ratio	−0.118	−0.500 to 0.302	0.573	−0.168	−0.512 to 0.222	0.383
C3a	0.397	−0.010 to 0.691	0.049	−0.027	−0.400 to 0.353	0.888
C4a	0.018	−0.390 to 0.421	0.930	−0.266	−0.584 to 0.122	0.162
TCC	0.194	−0.230 to 0.556	0.353	0.152	−0.238 to 0.500	0.431

CRP, C-reactive protein; PCT, procalcitonin; TNF-α, tumor necrosis factor alpha; IL-6, interleukin-6; IL-1β, interleukin-1 beta; IL-10, interleukin-10; C3a, complement 3a; C4a, complement 4a; TCC, terminal complement complex.

## 4 Discussion

In this secondary analysis, we investigated the interrelationship between the postoperative inflammatory response and vasoregulatory dysfunction in relation to the assumed modulatory effect of intraoperative HA treatment during OHT surgery. Intraoperative HA treatment was observed to result in a significantly lower rate of postoperative severe vasoregulatory dysfunction compared to controls, as evidenced by the vasopressor score analysis. This finding demonstrated a clear association with the mitigated PCT response associated with HA treatment and remained independent from cytokine and complement response.

### 4.1 Intraoperative hemoadsorption treatment associated reduced vasoregulatory dysfunction

CPB induced microvascular dysfunction is among the key elements of VS characterized by altered myogenic and vasomotor tone and generalized endothelial dysfunction affecting the responsiveness to endogenous vasoactive mediators, catecholamines and vasopressin (13, 14). The consecutive vasoregulatory dysfunction clinically manifests as significant hypotension and end-organ hypoperfusion promoting organ damage (14). The relationship between the quantity of summarized vasopressor dose and shock severity is confirmed by a substantial corpus of prior data (15). Moreover, in doses exceeding 0.3 μg/kg/min, norepinephrine can act as an independent contributor to unfavorable outcomes in septic shock (16, 17). In this context, the markedly reduced vasopressor score and incidence of severe vasoregulatory dysfunction observed in the HA group in comparison to controls show an influence of the HA treatment on the pathophysiological processes underlying the development of severe VS. Furthermore, the median vasopressor score of 19.7 in the HA-treated group can be interpreted as indicating a vasopressor requirement of <0.3 μg/kg/min of norepinephrine equivalent. This finding demonstrates a clear risk reduction in the high dose vasopressor associated adverse effects on postoperative organ dysfunction. Conversely, the elevated rates of postoperative acute kidney injury and renal replacement therapy, diminished hepatic bilirubin excretion, and prolonged periods of mechanical ventilation observed in the control group during the primary analysis of our proof-of-concept RCT, may be attributable to the cumulative impact of unstable post-transplant hemodynamics (median vasopressor score: 35.6) and the associated effects of excessive vasopressor doses (5). Over the last decade, several investigations have been conducted in the field of complex cardiac surgery to assess the impact of intraoperative HA treatment on clinical outcomes, including postoperative vasopressor need (6). Among these, four studies presented a consistent set of data on perioperative vasopressor doses (18–21). Similar to the results of our current analysis, these studies observed significantly lower vasopressor requirements in the interventional group compared to standard treatment in relation to the early postoperative period. Interestingly, one in four investigations demonstrated a significant benefit with regards to postoperative organ dysfunctions associated with HA treatment, while subsequent studies revealed only favorable trends in the same aspects (18–21).

### 4.2 Characteristics of the post-transplant inflammatory response in relation to hemoadsorption treatment

The activation of cytokine- and complement cascades induced by interactions with CPB and ischemia/reperfusion injury can progress to an excessive inflammatory response during the early postoperative course, which is presumed to contribute to the pathogenesis of multiple organ dysfunction associated with complex cardiac surgery (22). In the light of this pathophysiological background, a series of RCTs have been designed with the objective of evaluating the potential of intraoperative HA treatment in reducing perioperative cytokine and complement levels in a high-risk cardiac surgical population (23–27). It is noteworthy that none of the measured pro- and anti-inflammatory cytokines exhibited statistically significant differences between the HA-treated patients and the controls 24 h after surgery (23–27). In regard to the perioperative alterations in C3a and C5a levels, Gleason et al. observed comparable C3a concentrations across the study groups and significantly diminished C5a levels in the HA-treated group relative to the controls (25). The results of the current analysis are in accordance with the previously published data, as no differences were observed in the investigated cytokines or complement levels at the 24-h post-CPB measurement point between the study groups (Table 2). Furthermore, the post-transplant cytokine and complement profiles were also comparable between the HA-treated and control groups in the cluster of patients who developed severe vasoregulatory dysfunction (Table 3). However, both the pre-transplant immune priming (Table 1) and the post-transplant cytokine and complement responses were low in the study groups, according to the ranges of concentrations and the IL-6/IL-10 ratios. Accordingly, a review of the current and relevant previous data indicates that the role of the cytokine and/or complement response in the complex pathomechanisms of the postoperative vasoregulatory dysfunction associated with OHT surgery remains uncertain. Moreover, in evaluating the intensity of the post-transplant cytokine or complement response, it is essential to consider other concomitant factors, such as the potential modulating effect of the perioperative immunosuppressive regimen. In this context, Weis and colleagues demonstrated the clear suppressive and modulating effect of the stress dose hydrocortisone on the kinetics and early postoperative peaks of cytokines, especially IL-6 and IL-10, in their RCT of high-risk cardiac surgical patients (28). The intraoperative administration of an immunosuppressive dose of methylprednisolone in our study is at least one order of magnitude higher than the stress dose hydrocortisone, which is likely to contribute to the significantly reduced post-CPB levels of cytokines (i.e., TNF-α, IL-6, IL-1β, and IL-10). As the elimination potential of the hemadsorption is predominantly concentration dependent, this can account for the almost identical cytokine– and complement responses observed in both study groups, thereby demonstrating the absence of modulation of these markers in the low range by the intervention.

In contrast to the post-transplant cytokine and complement profile, a marked PCT response was observed in both the HA and control groups, confirming its median concentration and the rate of peak PCT > 1.0 μg/L to be significantly lower in the–HA treated patients than in the control group (Table 2). These statistically significant differences were also revealed in the cluster of patients with severe vasoregulatory dysfunction (Table 3). PCT has proved to be an early biomarker of the inflammatory response (29). As a part of the pro-inflammatory cascade, multiple tissues are induced to release PCT and an elevated level of PCT > 1.0 ng/mL can be indicative for postoperative organ dysfunction following cardiac surgery (29–31). However, in addition to the direct trigger of cytokines, the intraoperative hypoperfusion/reperfusion injury of the hepatosplanchnic region can also be distinguished as another significant trigger of the rapid and robust PCT release during cardiac surgery (32). As demonstrated in previous observational studies conducted in the field of complex liver surgery, there are explicit data for instant PCT release of the hepatosplanchnic region in relation to ischemia/reperfusion events (33, 34). This significant phenomenon is consistent with the findings of the study presented by Silomon and colleagues, who observed the highest PCT concentrations in the hepatic veins compared to the time-matched mixed venous or arterial levels among patients scheduled for elective coronary artery bypass graft surgery (32). In light of the pathophysiological characteristics of end-stage heart failure, reperfusion of the hepatosplanchnic region turns to clinical significance from the initial phase of CPB during OHT surgery. Consequently, it can be presumed that a significant hepatosplanchnic PCT induction occurs during CPB, which exerts a considerable influence on the post-CPB PCT kinetics. This component of the perioperative PCT response is potentially independent from the perioperative immunosuppressive treatment, and its kinetics and magnitude can be controlled effectively by the intraoperative HA treatment. This assumption is supported by the observations of this secondary analysis, which presented a significantly decreased PCT response in the hemoadsorption group in comparison with the control group.

Recent clinical data have demonstrated the presence of postoperative capillary leakage, increased requirements for fluids and vasopressors in patients with elevated PCT levels after CPB surgery, assuming a direct causative relationship between PCT and impaired microvascular integrity (35). The hypothesis that PCT can act as a mediator of microvascular dysfunction has been the subject of previous high-quality experimental investigations (35, 36). The findings of these studies indicate that PCT has a direct negative effect on endothelial cells, destabilizing endothelial adherens junctions and facilitating capillary leakage and vasoregulatory dysfunction (35, 36). The results of our secondary analysis corroborate those of the experimental findings. Although the vasopressor score did not show any relationship with the cytokine and complement levels in either the control or the HA groups, a moderate positive correlation was identified between the vasopressor score and the PCT concentration in the control group (*r*_S_: 0.605, *p* = 0.002, Table 4). Conversely, no statistical correlation was observed between PCT concentration and vasopressor score in the HA group. However, from a clinical perspective, the use of intraoperative HA treatment resulted in a markedly reduced post-transplant PCT response (Table 2), which can be linked to the less severe vasoregulatory dysfunction, and lower rate of post-transplant organ dysfunction observed in this group, as confirmed by the primary analysis of our proof-of-concept RCT. The results of this secondary analysis provide substantial support for the assumption that intraoperative HA treatment performed during OHT may beneficially modify the pathophysiological processes linked to OHT surgery and may be partly responsible for PCT induction, thereby reducing the PCT-related adverse effects. Consequently, this intraoperative intervention has the potential to prevent perioperative impairment of microvascular integrity and the development of severe vasoregulatory dysfunction and its associated end-organ dysfunction.

Among the potential clinical benefits of intraoperative HA treatment, the main basis of this intervention is to prevent or control the dysregulated hyperinflammatory response associated with complex cardiac surgery, particularly in selected high-risk patients (6). Considering the non-selective adsorption spectra of the HA technique, theoretically, this treatment method carries risk for adverse immunomodulation through the interactions with the actual pro-/anti-inflammatory balance and/or exogenous immunomodulatory agents (6). However, a recently published systematic review and a registry report did not confirm safety concerns related to intraoperative HA treatment, based on the incidence of reported unanticipated device-related adverse events (6, 37). In addition, in the primary analysis of our proof-of-concept RCT, we have also not registered intraoperative HA treatment–associated adverse effects such as postoperative bleeding, early sepsis and cardiac allograft rejection (5). Accordingly, the early inflammatory response, characterized by the 24-h cytokine and complement profiles in this secondary analysis, reinforces the clinical endpoints and the safety application of HA treatment during OHT surgery underlying by the nearly identical IL-6/IL-10 ratios in the study groups (Table 2).

## 5 Limitations

There are some limitations of this secondary analysis of our proof-of-concept RCT. In accordance with the inclusion criteria, the objective of our RCT was to recruit participants from the low-risk group of OHT recipients. With regards to the interrelationship between postoperative inflammatory response, vasoregulatory dysfunction and organ damage, an expanded patient profile encompassing high-risk OHT recipients could facilitate a more comprehensive delineation of the underlying pathomechanisms associated with OHT surgery. In order to facilitate a discriminative hemodynamic assessment and subsequent follow-up, and to minimize the bias generated by confounding factors that interfere with the perioperative vasopressor need, we applied protocolized extended hemodynamic monitoring and goal-directed algorithm-based management in the perioperative period of OHT. This is fully identical with the institutional OHT management protocol, which was described in detail earlier (5). Furthermore, the aforementioned limitations, in addition to the single–center design and sample size of this RCT, may have restricted the interpretation of our results to some extent. Nevertheless, in view of the significance of the findings of the present secondary analysis in shedding further light on the potentially beneficial impact of intraoperative HA treatment in the context of OHT, further investigation in this field is highly warranted.

## 6 Conclusion

The results of this secondary analysis of our proof-of-concept RCT indicate that intraoperative HA treatment during OHT is associated with a reduction in the severity of post-transplant vasoregulatory dysfunction and a lower risk of adverse effects related to excessive vasopressor doses as suggested by a significantly lower vasopressor score in the early postoperative period, compared to standard care. The present study did not identify any differences in post-transplant cytokine and complement profiles, nor did it confirm the dominant roles of cytokine and complement responses in the pathomechanisms of vasoregulatory dysfunction associated with OHT surgery. The results of this secondary analysis corroborate the deleterious correlation between elevated PCT levels and the severity of vasoregulatory dysfunction. The findings of this study indicate that intraoperative HA treatment exerts a beneficial modulatory effect on the postoperative PCT response in OHT recipients, which is directly associated with the significantly lower rate of post-transplant severe vasoregulatory dysfunction compared to controls.

## Data Availability

The original contributions presented in the study are included in the article/supplementary material, further inquiries can be directed to the corresponding author.
